# Surface Acoustic Waves-Based Molecular Recognition of a Collagen Receptor on Human Erythrocytes

**DOI:** 10.3390/ijms262311258

**Published:** 2025-11-21

**Authors:** Gevorg Ghukasyan, Narine Ghazaryan, Michael Torosyan, Naira Movsisyan, Ashot Meltonyan, Naira Ayvazyan

**Affiliations:** 1 Orbeli Institute of Physiology of NAS RA, Yerevan 0028, Armenia; gevorg.ghukasyan1@ysumail.am (G.G.); naringhazaryan@gmail.com (N.G.);; 2 Department Chemistry, Yerevan State University, Yerevan 0025, Armenia; mikael.torosyan.5@gmail.com; 3 Max-Planck-Institute of Experimental Medicine, 37075 Goettingen, Germany

**Keywords:** disintegrins, surface acoustic waves, erythrocyte ghosts, molecular dynamic simulation

## Abstract

Integrin-mediated binding is important for the metastatic dissemination of different types of cancer cells. Snake venom disintegrins obtustatin and echistatin are potent, irreversible, and selective inhibitors of α1β1 and αvβ3 integrins, respectively. Obtustatin is one of the shortest disintegrins yet described, containing 41 amino acids. It has a similar pattern of cysteines to the other disintegrin echistatin but with a KTS motif rather than a classic RGD in its active site. A surface acoustic wave biosensor was applied to prove the molecular recognition of disintegrins by their substrates. The human erythrocyte ghost cells were immobilized at the sensors to allow for the detection of kinetic binding constants of disintegrins compared to the surface of giant unilamellar vesicles (GUVs). Obtustatin binds to the erythrocyte ghost membrane with affinity in the mid-nanomolar range (2.32 × 10^–7^ M), and echistatin in the low micromolar range, which indicates specific molecular recognition for both disintegrins, but the higher response for obtustatin. The data directly confirm that disintegrins bind to the erythrocyte ghost membrane, thereby supporting the previously overlooked presence of integrins in red blood cell membranes.

## 1. Introduction

Integrins are a group of cell surface receptors that bind to various ligands and play key roles in facilitating interactions between cells and between cells and the extracellular matrix. These transmembrane glycoproteins consist of two noncovalently connected subunits, known as α and β. Each subunit spans the cell membrane once, with the majority of its structure located outside the cell and short tails extending into the cytoplasm. In mammals, 18 α and 8 β subunits have been described that can form 24 unique integrin receptors. A subset of these, known as collagen receptors, specifically bind to different types of collagens. For these collagen-binding integrins to be functional, subunits α1, α2, α10, or α11 must pair with the β1 subunit. Among them, the α1β1 integrin is recognized as a receptor that selectively binds to collagen type IV [1,2]. The integrin αvβ3 is expressed in endothelial cells, osteoclasts, melanoma, and other cell types [3,4], where it contributes to physiological functions such as blood vessel formation and tissue regeneration, and is also implicated in pathological conditions, including osteoporosis, cancer cell metastasis, adenoviral infections, and tumor-related angiogenesis [5,6].

Despite the long list of adhesion molecules expressed by erythrocytes, direct reports about the existence of integrins within the red blood cell membranes appeared only in the last few years. At the dawn of blood research, scientists believed that the erythrocyte membrane was composed of lipids and one single protein—hemoglobin—and adhesive and signaling proteins’ presence was unthinkable. Later, Pasini et al., made the identification of at least 534 different red cell membrane proteins [7]; recently, Wilson et al., used nano-LC-MS/MS to compare the proteome of adult and cord RBCs and reticulocytes, and 2838 unique proteins were identified, providing the most comprehensive compendium of RBC proteins to date [8,9]. However, the evidence concerning the presence of integrins remains elusive. Recent force microscopy-based (namely, AFM) measurements have demonstrated a single-molecule interaction between fibrinogen and an as-yet-uncharacterized receptor on the erythrocyte membrane, exhibiting a binding affinity marginally lower but quantitatively comparable to that of fibrinogen–platelet interactions, which was identified as “an αIIbβ3-related integrin” by its ability to inhibit eptifibatide (an αIIbβ3-specific inhibitor) [10]. The detection of αIIbβ3 integrin and non-integrin Ib glycoprotein on red blood cell membranes, which was reported by one group of scientists [11], was conclusively challenged by opposing data from another group [12]. Also, on the erythrocyte surface, integrins α4β1 (VLA-4) and CD36 (thrombospondin receptor) were among the first adhesion molecules identified, but the authors reported that they are both only expressed by reticulocytes but not by mature red cells [13].

Disintegrins are the large family of anti-adhesive proteins found in viper venom [4], whose structural and functional complexity contrasts with their small molecular size. Echistatin is a 49-amino-acid peptide belonging to the disintegrins family derived from *Echis carinatus* snake venom [14]. It acts as a highly effective inhibitor of platelet aggregation and cellular adhesion. Obtustatin, comprising only 41 amino acid residues, is one of the shortest disintegrins characterized to date [15]. It was purified from the venom of the Subcaucasian subspecies of the Levantine viper—*Macrovipera lebetina obtusa*—and was not found in other subspecies of this snake (*M. l. transmediterranea*, *M. l. turanica*) [16]. The first one contains the RGD tripeptide in its active site as the most known disintegrin, while the sequence of the integrin-binding loop of obtustatin is utterly different from other disintegrins and contains the KTS motif. The structure of obtustatin and echistatin with the same pattern of cysteines is highly homologous. However, specific interaction for obtustatin is directed to block α1β1 integrin in a highly selective manner [17,18,19], while echistatin is a potent irreversible αVβ3 integrin antagonist (Ki = 0.27 nM) [20,21,22].

A surface acoustic wave (SAW) biosensor was applied to detect disintegrin–erythrocyte ghosts binding on a molecular level. Based on the piezoelectric effects, variations in mass on the sensor surface can be identified by monitoring phase shifts in an imposed acoustic wave. Comprehensive explanations of the measurement principles, the foundational physical phenomena, and the mathematical frameworks employed to calculate kinetic binding parameters are available in references [23,24], while various application examples can be found in [25,26,27]. Surface acoustic wave (SAW) techniques have predominantly been employed in cellular studies involving bacteria, bacteriophages, yeasts [28,29,30], and mammalian cell interactions [31,32].

In the present study, the SAW method was applied to detect adhesion receptor-mediated interactions, i.e., between erythrocyte ghosts and echistatin or obtustatin. By immobilizing a membrane preparation of human red blood cells on the sensor surface, we were able to elucidate the binding kinetics of disintegrins to the erythrocyte membrane and thus confirmed the presence of α1β1 and αVβ3 integrins in red cell membranes. The following molecular dynamic simulation was applied to demonstrate the specificity of the obtustatin structure versus echistatin and the importance of the intracellular/extracellular ionic asymmetry for the disintegrin structural stability and, hence, their functional effectivity as an integrin blocker.

Nowadays, disintegrins are emerging as attractive pharmacological tools, not only as a potential therapeutic compound for treating some of the most aggressive forms of cancer due to their angiostatic activity, as reported earlier [33,34], but also as proposed new methods for developing inhibitors of red blood cell aggregation.

## 2. Results

### 2.1. Biosensor-Based Investigation of Disintegrins Binding

**Figure 1:**
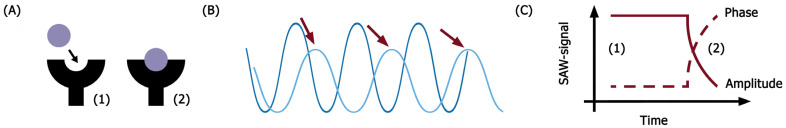
Surface acoustic wave biosensor principle of work. (**A**) Molecular interactions on the chip surface; (**B**) changes in surface acoustic wave shape during molecular interaction of receptors embedded on the chip surface with ligands; (**C**) the changes in signal detected and measured by the biosensor (1—amplitude; 2—phase). Red arrows demonstrate a shift of the signal.

To detect disintegrin/erythrocyte ghost binding at the molecular level, a surface acoustic wave (SAW) biosensor was used (Figure 1). The machines of SAW instruments use a detection technology resembling a highly sensitive “weight detector”. Binding processes are measured by real mass changes on the surface. Basically, the sensor is a chip, whereby the surface is excited to oscillate acoustically. Acoustic waves are generated in the piezoelectric material and propagate laterally. Response to changes in the surface-bound material and configuration is achieved through a proprietary method that produces a modified oscillation. This method and the high operation frequency significantly reduce distortion effects due to the complexity of the matrix; therefore, purification steps might be omitted [24]. The phase of the wave is shifted on mass changes. A change in amplitude indicates viscoelastic and conformational characteristics. Both effects can be differentiated and detected independently for interaction analysis [23].

**Figure 2:**
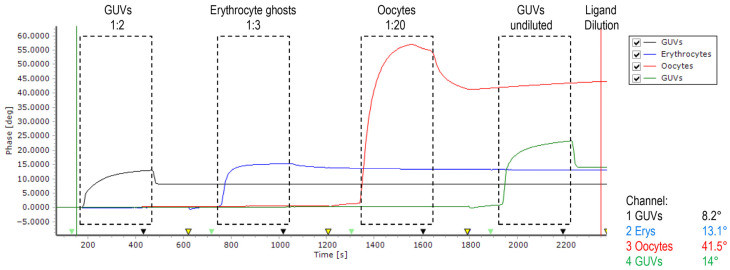
Phase versus time plot of sensor recorded during four subsequent measurement cycles. Between injections, the surface was regenerated using 10 mM glycine pH 2.2 (yellow arrows). The flow rate of running buffer PBS (pH 7.4 at 25 °C) is 20 μL/min. Baseline was stable except for oocytes, which continued to dissociate off the surface and were excluded from the experiments (see Figure S1 for data).

A similar amount of erythrocyte ghosts and GUVs were successfully immobilized on (1) positively poly-L-lysin charged 2D CM-dextran, (2) EDC/NHS-activated, negatively charged 2D CM-dextran, and (3) hydrophobic–CH3-SAM. The positively charged disintegrins were repelled from all charged surfaces; therefore, immobilization via hydrophobic interactions was chosen for the main core of experiments (Figure 2). Disintegrins were diluted in PBS to achieve similar immobilization levels; in a pre-experiment, all ligands were diluted 1:3, and then dilutions were adjusted. Experiments were performed in triplicate, starting with obtustatin, then echistatin, then vice versa. The baseline was stable; at immobilization of GUVs on a chip, a 14° phase shift was detected vs. 13.1° for the erythrocyte ghosts. In this set of experiments, GUVs formed from the phospholipid fraction of the bovine brain were used as a negative control for erythrocyte ghosts.

**Figure 3:**
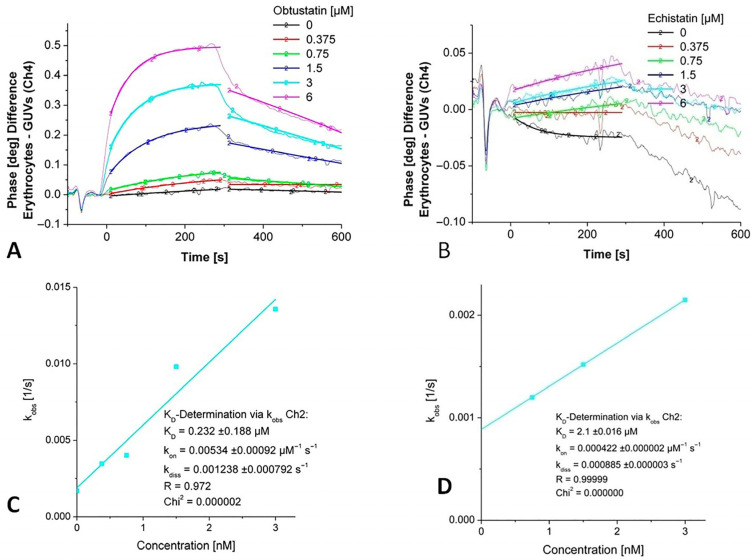
Detection of kinetic binding constants of echistatin and obtustatin binding to integrins in an immobilized membrane preparation (white erythrocyte ghosts). (**A**,**B**). Phase shift versus time diagrams for increasing concentrations of injected disintegrins echistatin and obtustatin. (**C**,**D**). A non-linear curve fitting in the area for the concentration depending on the association rate constant (k_obs_) vs. the area for the dissociation rate constant (k_off_) for obtustatin and echistatin, respectively.

Figure 3 shows the titration of the immobilized EG with increasing concentrations of disintegrins vs. unspecific binding to GUVs on a parallel channel. As is obvious, the highest difference is observable when mobile obtustatin interacts with erythrocyte ghosts, while the phase response for echistatin is remarkably lower. The phase response depends on the number of receptors on the cellular surfaces, and the number of integrins might be too low to generate a significant signal of the relatively small echistatin.

Based on the 1:1 binding model, a K_D_ value was calculated depending on the fit model applied for the interaction of disintegrins with integrins in the erythrocyte ghosts. Data were obtained from sensor signals after the administration of increasing concentrations of disintegrins using the monomolecular growth model. The observed rate constants (k_obs_) were determined from the rising phase of the sensor response curves for each analyte concentration and plotted against the corresponding disintegrin concentrations. A linear regression was performed on the dataset according to the equation

k_obs_ = k_on_ × c + k_off_

where k_on_ represents the association rate constant, k_off_ is the dissociation rate constant, and c is the concentration of the injected analyte (see Figure 3C,D). Kinetic analysis of obtustatin binding to erythrocytes yielded a dissociation constant K_D_ in the mid-nanomolar range, whereas echistatin binding displayed a K_D_ in the low micromolar range (Table 1).

### 2.2. Immunoblotting

**Figure 4:**
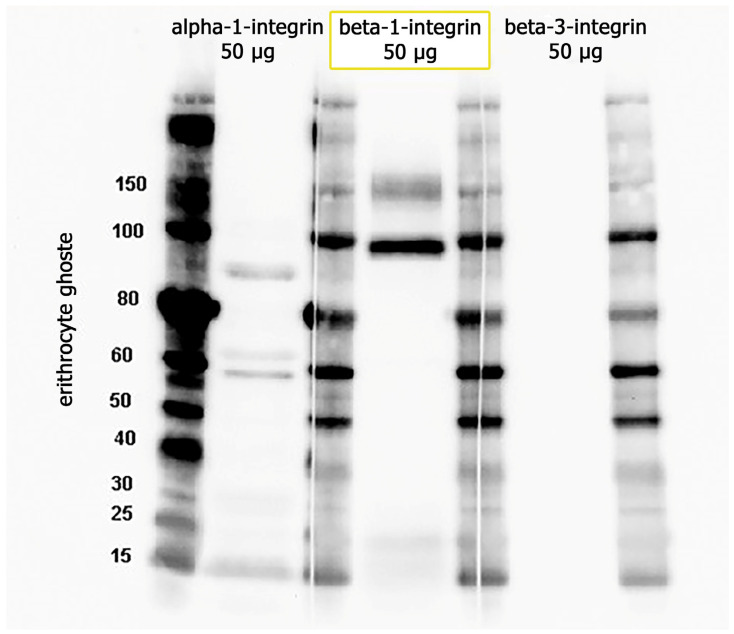
Proteins 50 (µg) from erythrocyte ghosts were separated in 4–12 NuPAGE Bis-Tris gels, followed by immunoblotting with antibodies against α1, β1, and β3.

To prove the presence of the integrins α1β1 and αVβ3 in the membrane of erythrocyte ghosts, we also performed Western blot analysis. In these experiments, we used α1, β1, and β3 primary antibodies (Table 3). From Figure 4, it is clear that beta-3 integrin is absent in the samples. For α1, we expected to detect the protein at 130 kDa (the predicted band size) or higher. It was contradictory, as we had two bands in the erythrocyte ghost sample. We used HeLa cells as a positive control for α1β1 integrin (while αVβ3 is known to have low expression in HeLa cells), and in this case, we detected a protein band at 150 kDa. This means that the protein undergoes glycosylation. By comparing the data obtained from HeLa cells (see Figure S2 for data) and erythrocyte ghosts, it seems that the α1 subunit may be damaged in the processing of ghost erythrocytes; it goes through hypotonic lysis and subsequent washing/resealing steps, disrupting the underlying cytoskeleton, which typically anchors integrin subunits to the membrane. The presence of β1 integrin (with two bands) on the surface of erythrocyte ghosts is prominent (Figure 4). Partially or not-glycosylated intracellular β1 in the endoplasmic reticulum (an 88–97-kDa precursor), as well as a completely glycosylated form at the cell surface (130 kDa), unlike other integrin subunits, exists regularly [35,36,37].

Proper glycosylation—carried out by specific enzymes within the structurally and functionally distinct compartments of the Golgi apparatus—is essential for β1′s activity. After maturation, β1 integrin is transferred to the plasma membrane, where it plays a key structural and signaling role by anchoring adhesion complexes to the actin cytoskeleton to facilitate both transmembrane and intracellular signal transmission.

### 2.3. Bioinformatics Processing and Molecular Dynamic Simulation

**Figure 5:**
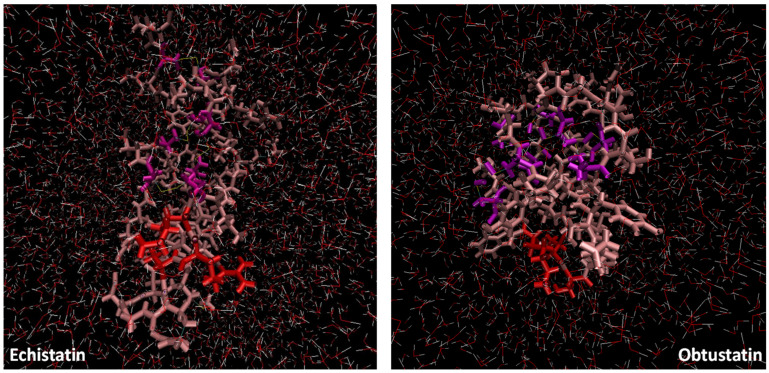
Representative snapshots of the simulation box of echistatin and obtustatin. The KTS and RGD motifs are colored in red, while the four cysteine linkages are colored in purple (the dimensions of the simulation boxes are the same).

The simplistic structure of these two disintegrins has been quite well investigated, but it still does not explain their high affinity and selectivity for integrins. A 100 ns long MD simulation of both disintegrins was performed in water at body temperature and at physiological pH to reproduce the normal physicochemical conditions (representative snapshots of simulation boxes are shown in Figure 5). These kinds of simulations, but with other more artificial parameters (20 °C and pH 3–4), were reported earlier [38]. The one other new approach in this MD simulation series was the comparative analysis of two disintegrins in the media with different types of ions, which is a situation mimicking the natural intracellular/extracellular asymmetry (the principal extracellular cation is sodium, while potassium ions have an intracellular concentration of 120 mM and an extracellular concentration of almost 30 times less—4 mM).

**Figure 6:**
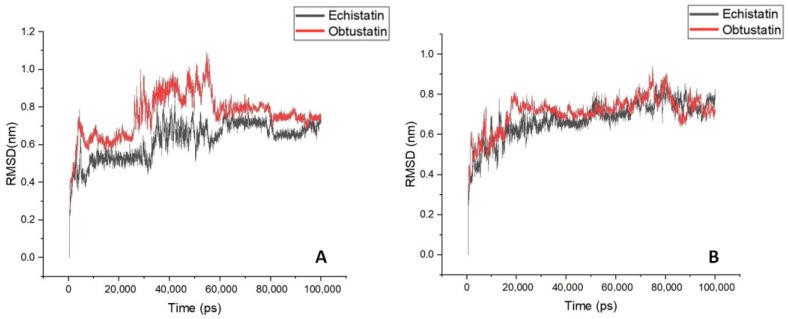
A time course of the backbone RMSD of disintegrin molecules is shown for aqueous solutions with potassium (Panel (**A**)) and sodium (Panel (**B**)) counterions addition for mimicking extracellular/intracellular ionic asymmetry.

**Figure 7:**
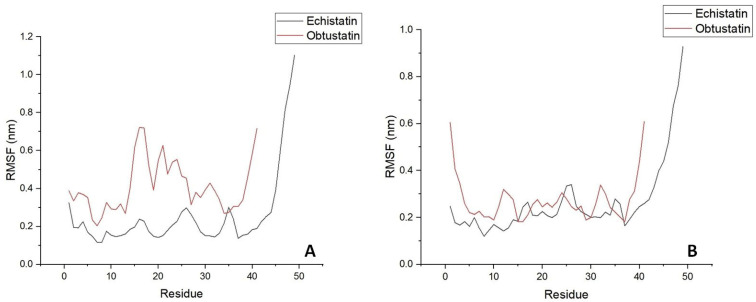
Time evolution of the secondary structure of disintegrin molecules; RMS fluctuations are shown depending on residue sequence number in water bulk of similar dimensions with potassium (Panel (**A**)) and sodium (Panel (**B**)) counterions addition for mimicking extracellular/intracellular ionic asymmetry.

In the Figure 6B plots, the root mean square deviation (RMSD) of the backbone atoms relative to the NMR structure is shown following the addition of sodium ions to the medium. In approximately 10 nanoseconds, the RMSD levels off at an average of about 0.7 nm. This deviation is distributed evenly across all residues, indicating no significant conformational shift in any particular region, especially in the case of echistatin. The two graphs presented in Figure 7B support this observation. The most pronounced fluctuations occur in the integrin-binding loop and the terminal ends (N- and C-termini).

To reveal the structural and specific dynamic behavior of the same disintegrins under different physicochemical conditions, a 100 ns long MD simulation was also performed, with the replacement of sodium ions by KCl at body temperature (36.6 °C) and pH 7.4. The backbone atom RMSD from this experiment is shown in Figure 6A. This time, we can observe much higher structure fluctuations both for the obtustatin and the echistatin, especially in the 30–55 ns timeframe. After ~60 ns, the RMSD values stabilized around an average of 0.7 nm, similar to that of the MD simulation with NaCl presence in the aqueous media. Interestingly, the root mean square fluctuation (RMSF) in the case of the potassium cations in the media shows that the obtustatin fold possesses distinct structural features compared to the echistatin: the differences that are not noticeable for the simulation mode with sodium ions (Figure 7A). While the structure of both disintegrins is well defined in the case of sodium ion presence, with approximately 70% of the residues in both conformers showing RMSF values below 0.4 Å (Figure 7B), obtustatin conformers in the presence of potassium ions display a higher overall RMSF of 1.1 Å concerning the mean structure. Only residues 1–15 and 27–38 remain well-ordered (RMSF < 0.4 Å), while regions 19–24 and 39–41, corresponding to the integrin-binding loop and the C-terminal tail, respectively, are more flexible. Notably, the integrin-binding loop of this disintegrin contains the KTS motif, which is critical for obtustatin to integrin α1β1 binding specificity and affinity.

In echistatin, as well as in obtustatin and other known disintegrin structures, the C-terminal tail lies close to and nearly parallel with the integrin specificity loop. This spatial arrangement is thought to be crucial for presenting key epitopes that trigger ligand-induced binding site (LIBS) formation [39]. In the case of obtustatin, this close positioning is partially stabilized by a disulfide bridge between cysteines at positions 19 and 36, a structural feature supported by experimental evidence [15].

While the specific role of obtustatin’s C-terminal region remains unexplored, the flexibility of both this segment and the integrin-binding loop—as well as their spatial proximity—may be key characteristics that facilitate the disintegrin’s ability to engage and inhibit integrin receptors. This interaction likely follows a “zipper” mechanism involving multiple contact points. Variations in the size, structure, and dynamics of the loop, as well as the unique positioning of the KTS motif, could significantly influence functional outcomes.

### 2.4. Raman Spectroscopy

Raman spectroscopy provided important insights into the secondary structures of various snake toxins [40]. Curiously, despite its early contributions, research using Raman scattering to study snake toxins has significantly declined since the 1990s. However, for the low-molecular-weight peptides from the snake venoms with their characteristic disulfide bonds and mostly undefined structures, Raman spectroscopy remains a powerful supportive biophysical investigational tool. It is well known that the amide I, II, and III vibrational modes could be sensitive indicators of a protein’s polypeptide secondary structure. Raman spectra of echistatin, a small RGD-containing protein, were very scrupulously investigated earlier [41], and here we have decided to compare its Raman scattering features with obtustatin spectra, where the most characteristic crucial motif is not an RGD but KTS. By its Raman spectra, echistatin indicates a considerable b-turn and b-sheet (20%) structure and the absence of an a-helix. Analysis of the amide-I′ and amide-III spectral features supports secondary structure estimations that align with circular dichroism data [41]. The absence of α-helical signatures, combined with pronounced carbonyl-stretching vibrations near 1660 cm^−1^ and 1680 cm^−1^, suggests a significant presence of β-turns and β-sheet configurations. Precise quantification of these elements is hindered by spectral overlap within the amide-I region. However, by referencing the C–H bending mode around 1440 cm^−1^ as an internal calibration point, the β-sheet content of echistatin can be approximated at 20% based on the intensity of the amide-III band observed at 1240 cm^−1^.

**Figure 8:**
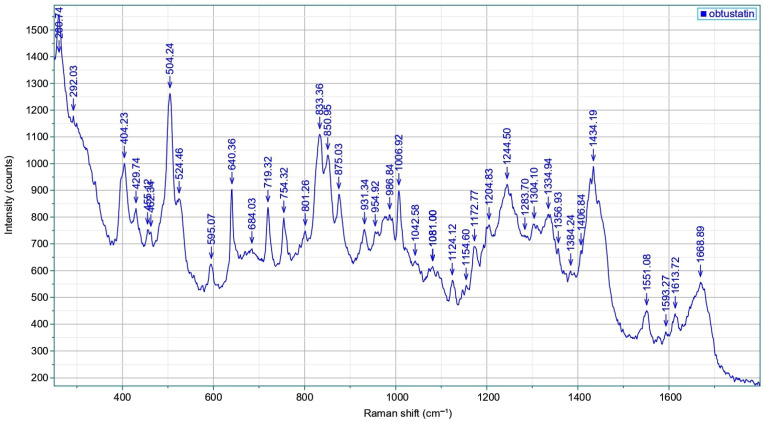
Raman scattering of the obtustatin: spectra reflect the sample composition, with several peak positions uniquely associated with particular amino acids and structural attributes. Specifically, the amide I (1600–1690 cm^−1^) and amide III (1230–1300 cm^−1^) regions reveal details about the secondary structure of proteins or peptides. The 810–870 cm^−1^ range features the tyrosine doublet, which indicates the local environment surrounding tyrosine residues. Meanwhile, the 490–550 cm^−1^ interval, known as the “S–S region,” assesses the spatial configuration of disulfide linkages (C–C–S–S–C). Also, the 1340–1350 cm^−1^ range is used to monitor the environment of tryptophan residue.

The Raman spectra with all the above-mentioned features and bands of obtustatin are shown in Figure 8 (circular dichroism data see in Figure S3). The amid-I band appears at 1669 cm^−1^, while carbonyl-stretching vibrations of the sidechains of aspartic and glutamic acids, which are well-pronounced in echistatin above 1700 cm^−1^ and the side-chain of the very characteristic band of phenylalanine 1008 cm^−1^, are absent here. Typically, α-helical conformations exhibit amide I’ absorption bands within the 1645–1650 cm^−1^ range, which are notably absent in this case. In contrast, β-sheet and β-turn structures display carbonyl stretching vibrations at higher wavenumbers, typically between 1660 and 1680 cm^−1^. The amide III region of obtustatin’s Raman spectrum was also measured: this band arises from a combination of N–H in-plane bending, as well as C–N and C–C stretching vibrations, and it is located in a spectrally congested region around 1244 cm^−1^. There are four disulfide bonds in obtustatin (similar to in echistatin); the Raman spectrum is observed at approximately 504 cm^−1^ and 524 cm^−1^, with an intensity ratio near 3:1. As described by Sugeta et al. [42], the lower-frequency mode corresponds to an all-gauche conformation. In contrast, the 524 cm^−1^ band reflects a trans-gauche-gauche configuration.

As we can see from the analysis of the amino acid compositions of disintegrins (Table 2) used for the present study, the differences between these two molecules are quite drastic on the level of the primary amino acid backbone, in spite of the secondary structure similarity. These data are in good agreement with earlier experimental data [39,41] and the results of the MD simulation. In contrast to the recently resolved crystal structure of the extracellular domains of integrin αvβ3 bound to an RGD-containing ligand, where arginine and aspartic acid sidechains (positions 24 and 26) form a complex web of polar interactions with residues in the αv β-propeller and β3 βA-domain [43], the key determinant for obtustatin’s ability to inhibit the α1β1 integrin–collagen IV interaction is supposed to be the central threonine within its KTS motif [15].

## 3. Discussion

Integrins are transmembrane heterodimeric receptors that function by either linking soluble or cell surface ligands, attaching cells to the extracellular matrix (ECM), or anchoring neighboring cells through their counterreceptors [44]. Beyond anchoring cells, they regulate essential behaviors like proliferation, spreading, growth, and programmed cell death. They also play key roles in immune responses and blood clotting. Functioning also as mechanoreceptors, integrins convert mechanical cues from the environment into intracellular signals that influence cell behavior [45]. Each integrin subunit contains an extracellular (the largest part of the subunit), a transmembrane, and a much smaller cytoplasmic domain. Integrins can shift between three structural forms: a bent shape, an extended form, and an extended state with an open headpiece. Their activation is a dynamic and tightly controlled process that is vital for functions like cell adhesion and migration, assembly of the extracellular matrix, and the transmission of mechanical signals. Furthermore, when ligands bind to integrins—a process known as “outside-in” signaling—it triggers alterations in the normal functioning of cytoplasmic enzymes such as kinases, GTPases, and phospholipases [46]. A highly structurally conserved von Willebrand factor A (vWFA) superfamily domain (so-called I domain) is present in some human α integrin subunits: α1, α2, α10, α11, αD, αE, αL, αM, and αX [47]. This domain is embedded between the second and third blades of the β-propeller and features a metal ion-dependent adhesion site (MIDAS), which facilitates ligand interaction [48,49]. MIDAS is critical in coordinating divalent metal ions, a necessary step for integrins to achieve a high-affinity binding state [50]. In contrast, α integrins that do not possess an I domain—such as α4, αIIb, and αV—rely on the βI domain to engage ligands. In conjunction with parts of the β-propeller, this domain also contains a MIDAS site [51,52]. Furthermore, the βI domain includes an adjacent site known as ADMIDAS, which binds calcium ions (Ca^2+^) that act as inhibitors by counteracting the activating effect of manganese ions (Mn^2+^) [47,53].

Disintegrins, small proteins commonly found in viper snake venoms, are notable for their capacity to interact with integrin receptors and influence key cellular processes, including inflammation, programmed cell death in endothelial cells, and the suppression of platelet aggregation [54]. These proteins typically feature specific amino acid motifs—such as RGD (Arg-Gly-Asp), MLD (Met-Leu-Asp), or K/RTS (Lys/Arg-Thr-Ser)—displayed prominently on surface loops that engage integrins on various cell types. Surrounding amino acids near the RGD sequence may contribute to an extended binding domain, enhancing integrin specificity and binding strength through structural complementarity [4]. Those with the RGD motif represent the most thoroughly characterized subgroup among disintegrins. Their distinct biological actions, particularly the capacity to block platelet aggregation—a key step in thrombus formation—have positioned disintegrins as promising candidates for developing antithrombotic therapies to treat or prevent disorders like stroke and deep vein thrombosis [5].

In this current investigation, we used two types of short disintegrins with quite well-known high selectivity to their respective receptors, which are αvβ3 and α1β1. Despite their similarity in size and structure, these two disintegrins are the representatives of totally different sub-types: the first one is a “classical” RGD-disintegrin, while the other one contains the KTS motif in its active site, which makes it a very potent and selective blocker of the collagen IV receptors. Collagen IV is a type of nonfibrillar collagen, meaning it does not assemble into fibrils but instead creates mesh-like structures within the extracellular matrix (ECM). In the body, collagen IV is a key component of the basal lamina—the layer of the basement membrane produced by epithelial cells—and is also found in capillaries. It serves as the primary structural framework of the basement membrane; so it is abundantly present in all types of blood vessels and plays a crucial role in angiogenesis [55].

Erythrocytes historically have been considered relatively passive bystanders in thrombosis. Arterial clots, commonly triggered by plaque rupture, are primarily composed of platelets and are referred to as “white” thrombi. In contrast, venous thromboembolism (VTE), encompassing deep vein thrombosis and pulmonary embolism, results from impaired endothelial function and reduced blood flow, which promote the formation of “red” thrombi composed primarily of fibrin and red blood cells. However, a range of clinical and population-based studies has linked both the quantity and quality of RBCs—such as variations in hematocrit and conditions like sickle cell disease, thalassemia, hemolytic anemias, and malaria—to increased risks of both arterial and venous thrombosis [56]. Emerging mechanistic research indicates that RBCs may actively contribute to thrombus development and stability, though many of these proposed pathways remain speculative and not yet fully elucidated.

The membrane’s flexibility and RBCs’ ability to maintain their morphology without a nucleus make them an exceptional model to study the mechanics of the cellular membrane [9,57]. The above-described biophysical approaches let us use the human red blood cells as the model membranes to identify specific integrins within the membranes and try to understand the mechanisms of the receptor–inhibitor interactions during disintegrin blockade. As we can conclude from the specific binding of the obtustatin with the erythrocyte ghost membrane in the 1:1 binding mode during the SAW experiments, the presence of the α1β1 can be accepted as proved, especially taking into consideration the data of immunoblotting, the results of which are correlated with the results of the SAW biosensor-based data. On the contrary, the integrin αvβ3 is rather absent in the membrane of RBC. The detected low micromolar range of interaction could be evidence of some unspecific binding, which could also be expected, considering the lower specificity and selectivity of echistatin to this integrin compared to the obtustatin affinity level to the collagen IV receptor. Interestingly, the β3 integrins with αIIbβ3 were implicated primarily in platelet aggregation, while for the αvβ3 integrins, no primary hemostatic function has been identified [55]. These findings suggest that RBCs may contribute to thrombosis pathophysiology and have the ability to directly interact with collagen IV receptors in the capillary and arteriole epithelial walls in the course of the red thrombi formation. Further investigations may reveal potential strategies for therapeutically targeting RBCs to reduce thrombosis, and disintegrins from viper venoms could play a leading role in such drug design [58].

However, with their simplistic structure, disintegrins are still very enigmatic molecules for understanding their potent ability to irreversibly block the integrin “outside-in” signaling function. Despite the growing number of publications investigating the potential use of snake venom disintegrins in angiogenesis-related conditions—such as cancer, eye diseases, tissue regeneration, wound healing, and cardiovascular disorders—the exact mechanisms of action remain elusive.

Such a non-conventional method as Raman spectroscopy can help better understand the dynamic of the disintegrins structure–function correlations based mainly on the NMR revealing of the structure of echistatin and obtustatin. The apparent similarity of not very well-defined structures with the same four disulfide bonds and active loop is completely leveled out with the differences of the amino acid sequences and, hence, their different dynamic abilities and bond vibrations in certain ionic media and pH. With the help of the MD simulation, we could demonstrate the crucial role of the K/Na asymmetry, which is naturally presented in living organisms, including humans. The disintegrins, which are acting mainly from the extracellular side of the membrane, are much more stable in the presence of sodium ions compared to potassium in the 36.6 °C conditions. Unfortunately, most volumes of the MD simulations based on the refined NMR structures never take into consideration either natural conditions surrounding the cell/ligand or the body temperature (we could find only the publications with room temperature as a standard approach for the simulations) [38,59].

In conclusion, despite the above-mentioned challenges, we demonstrated that a combination of non-conventional biophysical approaches, such as the SAW technique (love wave-based) and Raman spectroscopy, provides new insights into the erythrocyte membrane’s ability to interact with small snake venom proteins—disintegrins, known for their extreme selectivity in binding specific integrin receptors. While these findings hold much promise, we propose that future investigations also prove the presence of integrins in RBCs (at least α1β1 collagen IV receptors) and the active role of erythrocytes in red thrombi formation, two facts previously neglected. Ultimately, our data provide the first in silico evidence that extracellular/intracellular ionic asymmetry is essential for disintegrin structural stability, and that mimicking natural conditions (pH, body temperature) is crucial for MD simulations.

## 4. Materials and Methods

### 4.1. Materials

Obtustatin and echistatin disintegrins from *Macrovipera lebetina obtusa* and *Echis carinatus* snakes’ venom were purchased from Tocris Bioscience (London, UK). Antibodies for Western blotting were obtained from Abcam (Cambrige, UK) and GE Healthcare (Chicago, IL, USA). Packed red blood cells (RBCs) were obtained from the Haemathology Center after Prof. R. Yeolyan (Ministry of Health, Republic of Armenia).

### 4.2. Phospholipid Processing and GUV Formation

Lipid fractions were isolated from bovine brain (purchased from the local butcher company), according to the original Kates method [60]. The chloroform–methanol mixture was removed under reduced pressure, and then lipid residue was dissolved in nonane (3% solution). GUVs were prepared by the electroformation method, developed by Angelova and Dimitrov [61]. GUVs were formed in a temperature-controlled chamber that allows a working temperature range from 20 °C to 50 °C. GUVs were prepared using the following steps: ~2 µL of the phospholipid stock solution was spread on each of the two sample chamber platinum wires. The chamber was then dried for ~1 h to remove any remaining trace of organic solvent. The chamber and the buffer (Tris-HCl 0.5 mM, pH 7.4) were separately equilibrated to temperatures above the lipid mixture phase transition(s) (~10 °C over the corresponding transition temperature) and then 2 mL of buffer was added to cover the wires. Immediately after buffer addition, the platinum wires were connected to a function generator and a low-frequency AC field (sinusoidal wave function with a frequency of 10 Hz and amplitude of 2 V) was applied for 90 min. The mean diameter of these GUVs should be ~100 µm, as previously reported [62].

### 4.3. Erythrocyte Ghosts

Erythrocyte membranes were obtained by the method of Dodge, Mitchell & Hanahan [63], as described [64]. To obtain erythrocyte ghosts, after the last wash the RBC pellet was mixed with nine volumes of ice-cold lysis buffer (5 mM sodium phosphate) and stirred for 15 min at 0 °C. Subsequently, the unsealed erythrocyte ghosts were pelleted by centrifugation at 37,000× *g* for 10 min at 0 °C. After the centrifugation the ghosts were washed with ice-cold lysis buffer until residual hemoglobin was not visible. The RBC ghosts were suspended in about 0.5 volume of PBS and were kept frozen at −30 °C until use.

### 4.4. SAW Biosensor Measurements

Biosensor investigations were performed using a sam^®^-X system Surface Acoustic Wave biosensor supplied by SAW instruments (GmbH, Bonn, Germany), as described before [65]. Gold-coated sensors were incubated in a solution of 11-mercaptoundecanoic acid (10 mM) in ethanol abs. forming a self-assembled monolayer by binding of the sulfur headgroups to the gold surface (CH3-SAM Chip). The carboxyl groups were activated with 200 mM *N*-ethyl-*N*-(dimethylaminopropyl)-carbodiimide (EDC) and 50 mM *N*-hydroxysuccinimide (NHS) to bind disintegrins and/or vesicles. An excess of activated unreacted NHS esters was blocked by 1 M ethanolamine (pH 8.5). Running buffer was PBS containing 2.7 mM potassium chloride and 137 mM sodium chloride (pH 7.4 at 25 °C); flow rate was 40/20 μL/min. For regeneration of the chip surface, 10 mM glycine pH 2 was used.

The binding and dissociation curves generated at different ligand concentrations were fitted according to the “1:1 binding and residue” model using the OriginPro 7.5 (OriginLab Corp., Northampton, MA, USA) and FitMaster software 2.69 (SAW Instruments, Bonn, Germany) [66]. The observed association rate constants kobs for the five channels were averaged for each dilution, plotted against the analyte concentration, and linear regression was applied. The equilibrium dissociation constant (K_D_) was obtained according to the following equation:

K_D_ = k_off_k_on_^−1^;

where k_off_ [s^−1^] is the dissociation rate constant representing the intersection of the fitted line with the *y*-axis and k_on_ [conc^−1^ s^−1^]—the association rate constant representing the slope of the linear best fit.

### 4.5. Protein Extraction and SDS-PAGE

Erythrocyte ghosts were centrifuged for 15 min at 14,000 rpm to obtain proteins. Afterwards, the supernatant was removed, and the ghosts’ pellets were lysed in non-denaturing lysis buffer (1% Triton X-100, 50 mM Tris-HCl, 300 mM NaCl, 5 mM EDTA, and protease inhibitor cocktail (Roche, Basel, Switzerland)) for 30 min at RT.

Protein concentration determination was performed using a BCA (bicinchoninic acid assay) protein assay kit (ThermoScientific, Waltham, MA, USA) according to the manufacturer’s instructions and was calibrated with BSA by measuring the absorbance at 562 nm and comparing with protein solutions of known concentration [67].

NuPAGE reducing agent (Invitrogen, Waltham, MA, USA), and NuPAGE LDS sample buffer (Invitrogen, Waltham, MA, USA) were added to the samples in 1:10 and 1:4 ratios to the final volume, respectively, in order to disrupt protein secondary structure and allow proteins to migrate depending on the mass to charge ratio during sodium dodecyl sulphate polyacrylamide gel electrophoresis (SDS-PAGE). Then the reduced samples were incubated at 70 °C for 10 min.

The denatured samples (50 µg) were resolved on 4–12% NuPAGE Novex Bis-Tris Mini Gels (Invitrogen) at 200 V. To identify the molecular weight of protein of interest; the ColorPlus prestained protein ladder (BioLabs, San Diego, CA, USA) was used.

### 4.6. Immunoblotting

Once protein samples were separated by SDS-PAGE, the proteins were transferred onto a nitrocellulose membrane (NC) (GE Healthcare, 0.45 µm) using a tank (wet) electro-transfer procedure. The following buffer was used for the transfer: 10 mM NaHCO_3_, 3 mM Na_2_CO_3_, 0.01% SDS, and 20% methanol. To allow the proteins to move from the SDS polyacrylamide gel towards the NC membrane, the voltage gradient was applied starting from 10 V and increasing it every 10 min by 10 V steps until 50 V, and the transfer was continued for 40 min at 50 V. After the transfer accomplishment, the NC membranes were rinsed with deionized water and dried for 2 h at RT. The membranes were rehydrated with deionized water and treated with Pierce Western blot signal enhancer (ThermoScientific, Waltham, MA, USA) according to the manufacturer’s instructions. The membrane was then incubated for 2 h in blocking buffer containing 0.1% casein (Roche, Basel, Switzerland), to prevent non-specific binding of the antibody probes to the membrane surface.

Then the NC membranes were incubated with primary antibody diluted in the blocking buffer overnight at 4 °C (Table 3). The membranes were incubated for 45 min with horseradish peroxidase (HRP) linked secondary antibody diluted in blocking buffer (Table 3) after 5 washes with deionized water and 5 min incubation with TBS-T. Then the membranes were washed 7 times with deionized water and incubated for 5 min in TBS-T. At the end, the chemiluminescent HRP substrate (Millipore, Burlington, MA, USA) was applied to the blots for 5 min, initiating the chemiluminescence reaction catalyzed by the peroxidase conjugated to the secondary antibody. The emitted light was detected with a ChemiDoc XRS system (Bio-Rad, Hercules, CA, USA); the image acquisition was conducted using Quantity One 1-D Analysis software v4.6.9 (Bio-Rad, Hercules, CA, USA). FIJI-ImageJ 2.0.0-rc-2 software was used for image processing analysis [68].

### 4.7. Raman Spectroscopy

Raman detections were excited by a 785 nm laser, and all spectra were recorded using a confocal Raman microscope (XploRA PLUS, Horiba Scientific, Palaiseau, France). Measurements were conducted with the use of a 50× objective, as well as one spot measuring method. During measurements, for the best relation between resolution and sensitivity, the optimum parameters of the spectrometer were selected. The IR laser was chosen to avoid the luminescence and 1200 g mm^−1^ grating. The instrument was calibrated against silicon at peak position 520 cm^−1^ under 532 nm/632 and nm/785 nm laser excitations. Data acquisition conditions for Raman measurements: spectral resolution, 3.0 cm^−1^; integral times of 3 s, and 10× accumulations. All Raman spectra were taken on solid samples.

### 4.8. MD Simulation

MD simulations of obtustatin and echistatin were performed in water using the GROMACS software 2021.4-2 package and the OPLS-AA force field [69]. Water was represented with the simple point charge model. The starting coordinates for the MD simulation of obtustatin and echistatin were taken from the 2D-NMR structure (pdb codes: 1MPZ and 2ECH, respectively). For each protein, one molecule was placed in a periodic cubic box large enough to contain the protein and at least 1 nm of solvent on all sides. A standard protocol was adopted to initiate the simulations. Periodic boundary conditions were applied to the simulation box, and the long-range electrostatic interactions were treated with the particle mesh Ewald method [38] using a grid spacing of 0.12 nm combined with a fourth-order B-spline interpolation to compute the potential and forces between grid points. The real space cut-off distance was set to 1.0 nm. The protonation state of the N-terminal and C-terminal ends of the proteins and of the sidechains were consistent with the physiological pH (pH 7.4) used in the experiments. Negative counter ions (KCl and NaCl) were added to the simulation box of obtustatin and echistatin, respectively, to ensure electrical neutrality and natural extracellular/intracellular ionic asymmetry.

The simulation lengths of the two systems were 100 ns, and 310 K (body) temperature conditions were applied. The first 10 ns of the MD trajectory of obtustatin and echistatin was excluded from the analysis to ensure proper equilibration.

## Figures and Tables

**Table 1:** Kinetic binding constants of obtustatin and echistatin binding to integrins in an immobilized erythrocyte ghost membrane.

	Obtustatin	Echistatin
**k_on_**	(5.34 ± 0.9) × 10^3^ M^−1^ s^−1^	(4.22 ± 0.002) × 10^2^ M^−1^ s^−1^
**k_off_**	(1.238 ± 0.8) × 10^−3^ s^−1^	(8.85 ± 0.03) × 10^−4^ s^−1^
**K_D_**	(2.32 ± 1.88)× 10^−7^ M	(2.1 ± 0.016) × 10^−6^ M

**Table 2:** Amino acid compositions of disintegrins obtustatin and echistatin.

Echistatin (P17347)----ECESGPCCRNCKFLKEGTICKR—ARGD-DMDDYCNGKTCDCPRNPHKGPAT Obtustatin (P83469)---CTTGPCCRQCKLKPAGTTCW-----KTS--LTSHYCTGKSCDCPLYPG--------		
**Name**	**Echistatin**	**Obtustatin**
Glutamic acid	3	0
Cysteine	8	8
Serine	1	3
Glycine	5	4
Proline	4	4
Arginine	4	1
Asparagine	3	0
Lysine	5	4
Threonine	3	7
Leucine	1	3
Isoleucine	1	0
Alanine	2	1
Phenylalanine	1	0
Aspartic acid	5	1
Methionine	1	0
Tyrosine	1	2
Histidine	1	1
Glutamine	0	1
Tryptophan	0	1
Total residues	49	41
References for amino acids	[14]	[15]

**Table 3:** List of primary and secondary antibodies used for immunoblotting.

Cat. Number	Antibody	Source	Dilution	Manufacturer
ab75872	Anti-Integrin beta 3 antibody	Rabbit monoclonal	1:1000	Abcam
ab179471	Anti-Integrin beta 1 antibody	Rabbit monoclonal	1:1000	Abcam
ab181434	Anti-Integrin alpha 1 antibody—C-terminal	Rabbit polyclonal	1:1000	Abcam
NA934-100UL	ECL Anti-Rabbit IgG, HRP Linked	Donkey/polyclonal	1:10,000	GE Healthcare

## Data Availability

The original contributions presented in this study are included in the article/Supplementary Materials. Further inquiries can be directed to the corresponding author.

## Supplementary Materials

The following supporting information can be downloaded at: https://www.mdpi.com/article/10.3390/ijms262311258/s1.

## Abbreviations

The following abbreviations are used in this manuscript:

GUV	giant unilamellar vesicles
SAW	surface acoustic wave
RBC	red blood cells
AFM	atomic force microscopy
KTS/RGD	lisine-threonine-serine/arginine-glycine-aspartic acid
SDS-PAGE	sodium dodecyl sulphate polyacrylamide gel electrophoresis
RMSD	root mean square deviation
RMSF	root mean square fluctuation
LIBS	ligand-induced binding site
vWFA	von Willebrand factor A
MIDAS	metal ion-dependent adhesion site
VTE	venous thromboembolism
