# Supplementary figures and images for: Surface Acoustic Waves-Based Molecular Recognition of a Collagen Receptor on Human Erythrocytes
Source: Int J Mol Sci. 2025 Nov 21;26(23):11258. doi: 10.3390/ijms262311258 (PMC12692147; doi:10.3390/ijms262311258)

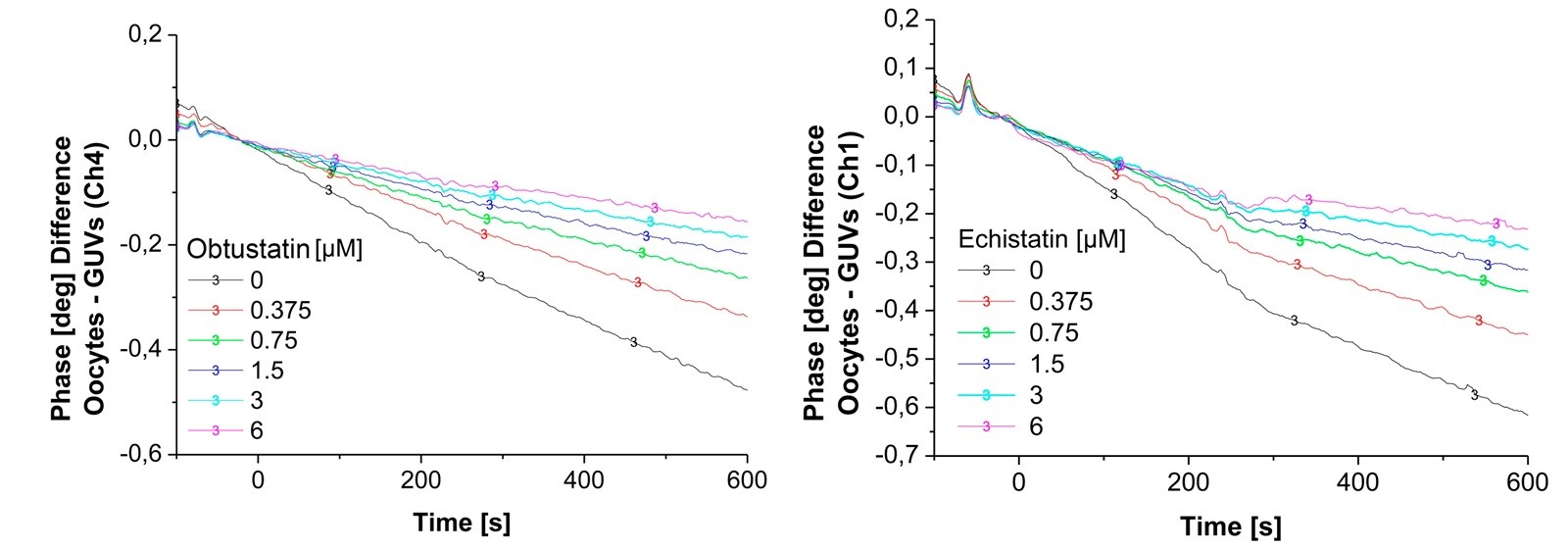

Supplement: Supplementary file 1 [file ijms-26-11258-s001.zip › Suppl. Figure S1.jpg]

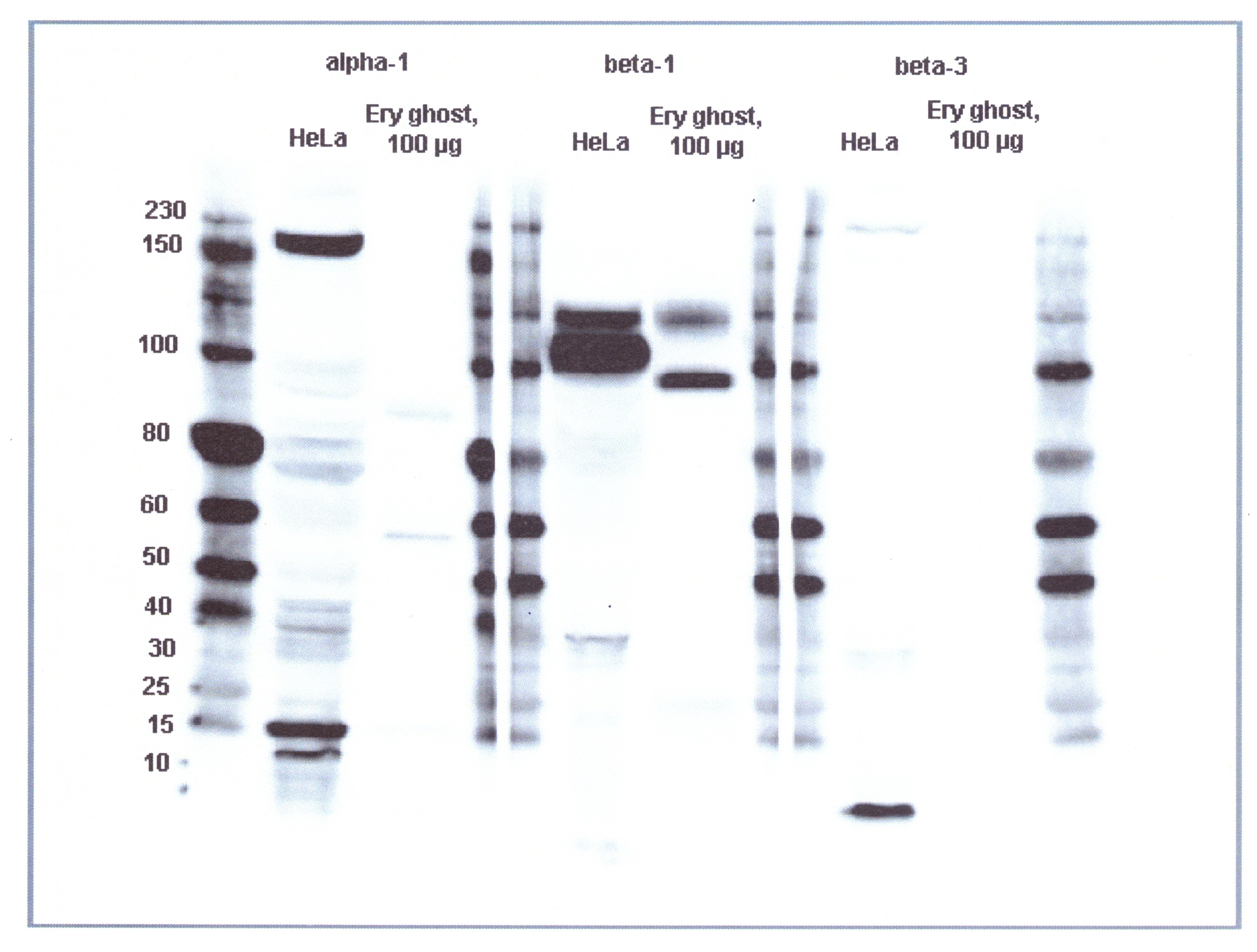

Supplement: Supplementary file 1 [file ijms-26-11258-s001.zip › Suppl. Figure S2.jpg]

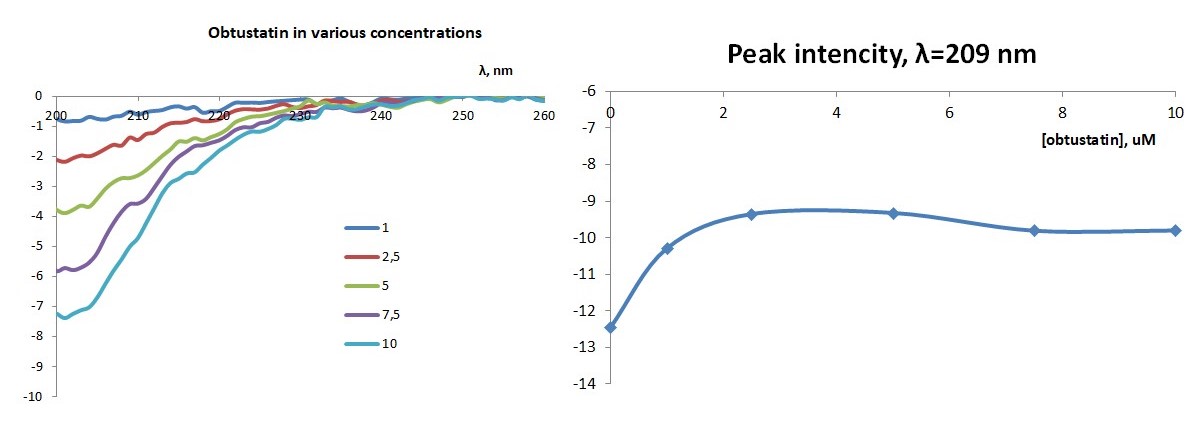

Supplement: Supplementary file 1 [file ijms-26-11258-s001.zip › Suppl. Figure S3.jpg]
